# Effects of Prosthetic Rehabilitation on Temporomandibular Disorders: Protocol for a Randomized Controlled Trial

**DOI:** 10.2196/33104

**Published:** 2021-12-24

**Authors:** Saranya Sreekumar, Chandrashekar Janakiram, Anil Mathew

**Affiliations:** 1 Department of Prosthodontics and Implantology, Amrita School of Dentistry Amrita Vishwa Vidyapeetham Kerala India

**Keywords:** orofacial pain, joint pain, prosthesis, edentulism, TMD, temporomandibular disorder, prosthetic rehabilitation

## Abstract

**Background:**

Loss of teeth or occlusal imbalance is one of the proposed dental risk factors for temporomandibular disorders (TMDs). Losing some non–free-end teeth cause the original occluding tooth/teeth to supraerupt from the original upright position and causes neighboring tooth/teeth to shift in an angle, causing biomechanical imbalance on the mandible. Based on these sequelae, rehabilitation of missing teeth is the first step in managing TMD in edentulous patients. Even though the prevalence of TMD in association with edentulism and in rehabilitated patients has been increasing, proper guidelines for the management of such cases have not been established. This study describes the protocol to analyze the effect of prosthetic rehabilitation on patients with TMD.

**Objective:**

This study aims to determine the effectiveness of prosthetic rehabilitation in the reduction of pain in edentulous patients with TMD and to determine the effect of the span of edentulism, the number of quadrants involved, pathological migration, the type of Kennedy classification, and the prosthetic status on temporomandibular joint dysfunction signs and symptoms.

**Methods:**

In this randomized controlled trial, 300 patients diagnosed with TMD will be grouped into one of the three interventional groups based on the type of their edentulous state. The interventional groups are (1) partially edentulous arch: Kennedy Class I and II (prosthetic rehabilitation without splint); (2) partially edentulous arch: Kennedy Class III and IV (prosthetic rehabilitation with a splint); and (3) completely edentulous arches (prosthetic rehabilitation without splint). All three of the mentioned interventional groups have corresponding control groups that will receive symptomatic treatment and comprehensive counseling. The measured primary outcomes are pain and electromyogram, and the secondary outcomes include pain drawing, Graded Chronic Pain Scale, Jaw Functional Limitation Scale, Oral Behaviours Checklist, depression, physical symptoms, and anxiety. The outcome measurements will be recorded at baseline and at the end of 24 hours, 7 days, 28 days, and 3 months.

**Results:**

Ethical approval was obtained from the Institutional Review Board of Amrita Institute of Medical Sciences, Kochi, India. Study participants’ recruitment began in May 2021 and is expected to conclude in March 2023. This clinical trial protocol was developed based on the SPIRIT (Standard Protocol Items: Recommendations for Interventional Trials) 2013 Statement.

**Conclusions:**

The purpose of this study is to gather data on prosthetic rehabilitation as a treatment for TMD. Obtaining this goal will aid in the development of evidence-based therapy protocols for prosthetic rehabilitation in TMD management.

**Trial Registration:**

Clinical Trials Registry - India CTRI/2020/06/026169; http://ctri.nic.in/Clinicaltrials/pdf_generate.php?trialid=42381

**International Registered Report Identifier (IRRID):**

DERR1-10.2196/33104

## Introduction

### Background

Temporomandibular disorders (TMDs) are defined by the American Academy of Orofacial Pain as “a collective term that embraces a number of clinical problems that involve the masticatory muscles, the temporomandibular joint, and the associated structures” [1]. TMDs are characterized by clinical signs such as muscle and/or temporomandibular joint tenderness; temporomandibular joint sounds (clicking, popping, or grating) while opening or closing the mouth or while chewing; and restriction, deviation, or deflection of the mouth while opening or closing [1].

The sum or exacerbation of these signs and symptoms eventually limits or even disables individuals in their physiological activities [2]. Between 40% to 75% of the population has at least one TMD sign such as noise in the temporomandibular joint, and 33% have at least one symptom, facial pain, or temporomandibular joint pain [3]. Between 65% to 85% of humans experience some symptoms of temporomandibular joint dysfunction at some time during their life, and 5% to 7% of the whole population requires treatment to decrease the symptoms of TMD [4]. Due to the high variability in the presentation of this disorder, TMD is diagnosed by its associating signs and symptoms [5,6].

TMD have been identified as a major cause of orofacial pain of nondental origin [7]. The World Health Organization has emphasized the importance of being free of chronic orofacial pain as a clear prerequisite for oral health as well as the negative effect of functional problems, such as chewing and eating, on the individual’s well-being and daily living, making them determinants of oral and general health [8]. Individuals with TMD symptoms have been found to seek different care providers and use the health care system to a greater degree as well as being more frequently on sick leave than people without these conditions [9]. Patients with TMD consequently experience a considerable negative effect on their quality of life [10].

TMDs have a complex and multifactorial etiology. One contributing factor that has been debated for years is the “occlusal condition” of the patient [10]. Loss of teeth or occlusal imbalance is one of the proposed dental risk factors for TMD. These dental factors include posterior crossbite, overjet/overbite greater than 5 mm, centric relation/maximum intercuspal sliding greater than 2 mm, edge-to-edge bite, sagittal relation class III, anterior open bite, and missing teeth [11,12]. Teeth are the most important components of the masticatory system with a close relationship with the temporomandibular joint and masticatory muscles. Any change to their normal functioning can induce pathological changes in the temporomandibular joint. *Missing posterior teeth* has been shown to have varied effect on the incidence of TMD [13-15].

Loss of teeth without replacement, especially at an early age, often causes the original occluding tooth/teeth to supraerupt from the original upright position and causes the neighboring tooth/teeth to shift in angle. Once the tooth/teeth begin to shift in angle, the vector of force tends to increase tooth-/teeth-tilting, thus imposing a different biomechanical effect on the mandible [16]. There is gender predilection, wherein the female mastication system may have less ability to withstand harmful stimulation from abnormal occlusion compared to counterparts, and thus, females may be more susceptible to TMD than men [17].

Little has changed in terms of study designs for temporomandibular joint research in the last decade, and treatment for patients with severe TMD remains controversial [18]. Following loss of occlusion due to tooth loss, there are secondary changes in the temporomandibular joint, which may accentuate further signs and symptoms [19]. The scientific community is uncertain about occlusion as the dominant cause of TMD nor do they justify prosthetic rehabilitation as the primary treatment modality for the management of TMD. The irony is that even though there is lack of evidence for loss of occlusion as a cause of TMD, the standard protocol in the management of TMD with edentulism is prosthetic rehabilitation.

There are still disagreements over the most effective and cost-effective treatment approach that can be widely distributed and used [20]. It is based on the clinical experience that, as the teeth are the most important components of the masticatory system and as they have a close relationship with the temporomandibular joint and masticatory muscles, any change to their normal functioning can induce pathological changes in the temporomandibular joint. Although prosthetic rehabilitation is the primary step in managing TMD, it is hardly justified with the evidence in the literature. With these gaps in knowledge regarding occlusion as a causative factor for TMD in the literature, we designed this trial to generate evidence regarding prosthetic rehabilitation for the management of TMD. Achieving this objective will help to develop the evidence for a treatment protocol for prosthetic rehabilitation in the management of TMD.

There are 3 intervention groups and their corresponding 3 control groups. The study has prosthetic rehabilitation with or without a splint (intraoral appliance) as the intervention. The comparator agent is symptomatic treatment and comprehensive counselling. If the patient is diagnosed with jaw joint disorder and has missing teeth, and if they are willing to take part in the study, they will be grouped into one of the three interventions or one of the three control groups based on the type of the toothless condition.

### Objectives

#### Research Question

Can prosthetic rehabilitation of edentulous patients with TMD decrease the pain symptoms of TMD?

#### Hypothesis

Prosthetic rehabilitation of edentulous patients will reduce pain symptoms of TMD as compared to patients without rehabilitation.

#### Primary Objective

The primary objective is to determine the effectiveness of prosthetic rehabilitation in the reduction of pain in edentulous patients with TMD.

#### Secondary Objective

The secondary objective is to determine the effect of the span of edentulism, the number of quadrants involved, pathological migration, the type of Kennedy classification, and the prosthetic status on temporomandibular joint dysfunction signs and symptoms.

### Trial Design

This trial is designed as randomized, as controlled, with a parallel arm, as blinded for outcome measurement, and with adaptive trial design.

## Methods

### Study Setting

The trial will be conducted at Amrita School of Dentistry, AIMS, Kochi and Amrita Urban Health Centre, Kaloor, Kochi, Kerala, India.

### Study Population

Partially and fully edentulous individuals reporting to the centers for routine dental care will be recruited for the study with their consent. Patients belonging to any gender, with/without prosthetic rehabilitation, and with/without any complaint of TMD with varied edentulous span will be included in the study.

### Clinical Definition

Patients presenting with pain in the jaw or temple area, pain or stiffness in the jaw, or pain on functional movements of the jaw (International Classification of Diseases, Tenth Revision, Clinical Modification Code M26.60). These symptoms are evaluated by the TMD screening questionnaire. Individuals giving a positive to any of the 3 screening questions in relation to the Diagnostic Criteria for Temporomandibular Disorders (DC/TMD) will be recruited to the study. The TMD pain screener has showed high sensitivity and specificity for detecting TMD pain [21].

### Eligibility Criteria

#### Inclusion Criteria

The following inclusion criteria will be used:

- Individuals within an age limit of 20-80 years
- Individuals completely or partially edentulous for a minimum period of 0-10 years
- Completely or partially nonrehabilitated edentulous individuals
- Patients who show signs and symptoms of myalgia of facial muscles
- Individuals who understand the importance of prosthetic rehabilitation and oral splints for TMDs
- Individuals who are willing to report at the required intervals for evaluation

#### Exclusion Criteria

The participant may not enter the trial if *any* of the following apply:

- TMD associated with macrotrauma of the head
- TMD associated with inflammatory/infectious/congenital disorders of the temporomandibular joint
- Individuals with Class II, Class III, or transverse malocclusion
- Individuals who refuse dental treatment
- Individuals with skeletal or dental developmental abnormalities and serious chronic medical conditions
- Any other significant disease or disorder that, in the opinion of the investigator, may either put the participants at risk because of participation in the trial or may influence the result of the trial or the participant’s ability to participate in the trial

### Patient Selection

Partially and fully edentulous individuals reporting to the centers will be recruited for the study with their consent. Patients belonging to any gender, with/without prosthetic rehabilitation, and with/without any complaint of TMD with varied edentulous span will be included in the study. After obtaining an informed consent, these patients will be screened using the DC/TMD screener questionnaire. The patients who are screened positive and satisfy the inclusion/exclusion criteria will be recruited to the study. They will undergo detailed oral examination and the TMD examination using the DC/TMD Axis I and Axis II questionnaire. The electromyography (EMG) reading of these patients will also be recorded. These patients will be allocated to the specific arms of the study based on their dentulous state.

### Interventions

**Figure 1:**
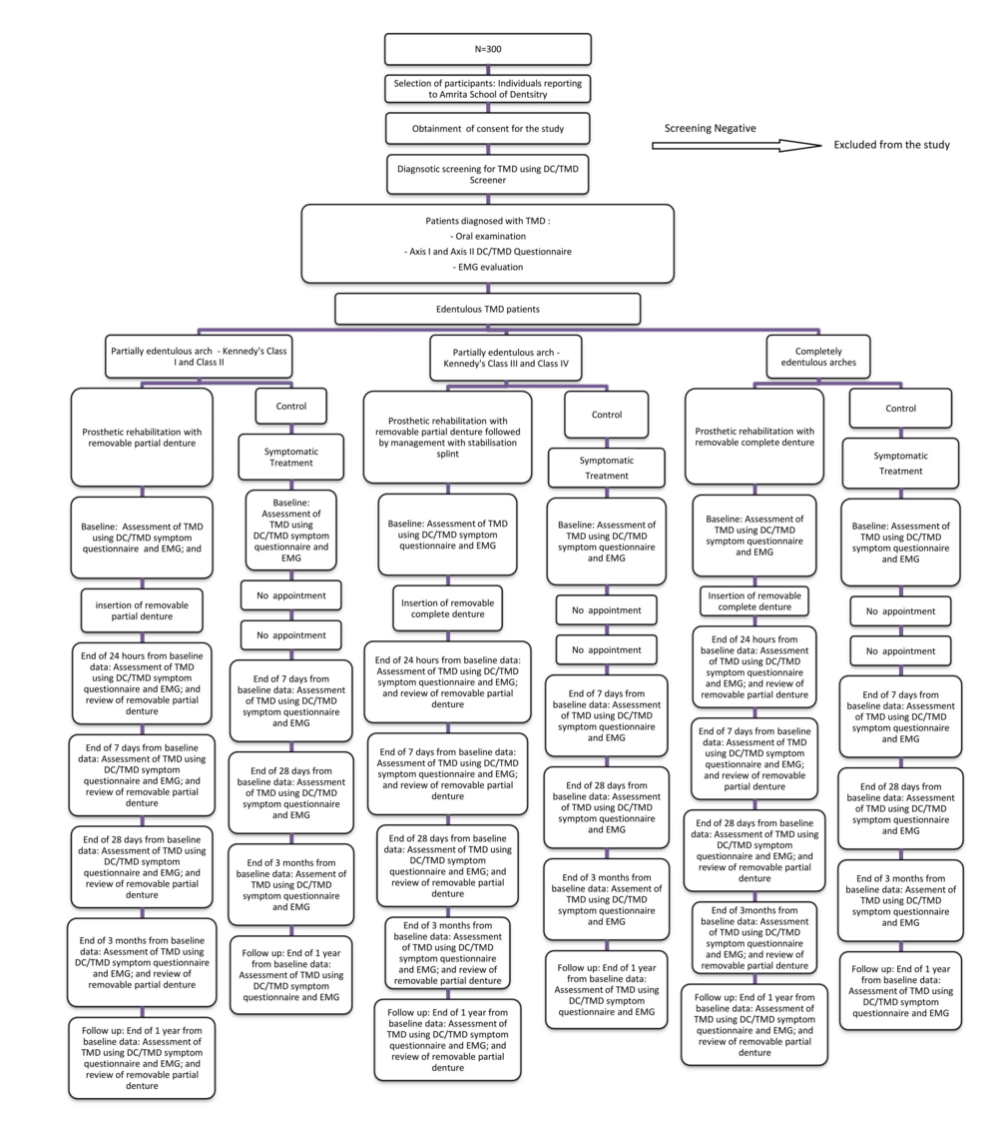
Flowchart showing study design. DC/TMD: Diagnostic Criteria for Temporomandibular Disorders; EMG: electromyography; TMD: temporomandibular disorder.

The participants will be grouped into 3 interventional and 3 control groups based on their state of edentulism (Figure 1; higher-resolution version in Multimedia Appendix 1). The interventional groups are:

1. Partially edentulous arch—Kennedy Class I and Class II: Kennedy Class I describes a patient who has edentulous areas bilaterally, posterior to the remaining natural teeth. Kennedy Class II describes a patient who has a one-sided edentulous area, posterior to the remaining natural teeth. The participants of this group will receive mouth preparation, prosthetic rehabilitation with removable partial denture, and comprehensive counseling (n=50).
2. Partially edentulous arch—Kennedy Class III and Class IV: In Class III the edentulous area has teeth located both anteriorly and posteriorly to it. In Class IV, there will be a single but bilateral (crossing the midline) edentulous area located to the anterior of the remaining natural teeth. The participants of this group will receive mouth preparation and prosthetic rehabilitation with removable partial denture followed by management with stabilization splint and comprehensive counseling (n=50).
3. Completely edentulous arches: The participants of this group will receive prosthetic rehabilitation with removable complete denture and comprehensive counseling (n=50).

Each of the 3 mentioned interventional groups will have their corresponding control groups with 50 participants in each control group. The participants in all the 3 control groups will receive symptomatic treatment and comprehensive counseling.

### Outcomes

The outcome measurements will be made by an examiner calibrated according to the guidelines of DC/TMD criteria. The DC/TMD instruction video has been peer-reviewed and published [22]. Self-study guidelines and description of supervised skill development have also been described. The outcome measurements will be made at baseline and at the time points of 24 hours, 7 days, 28 days, and 3 months.

#### Primary Outcome Measurements

##### Pain

Pain will be measured using the visual analog scale (VAS) for pain. The pain VAS is a unidimensional single item measure of pain intensity [23], which has been widely used in diverse adult populations. Using a ruler, the score is determined by measuring the distance (mm) on the 10-cm line between the “no pain” anchor and the patient’s mark, providing a range of scores from 0 to 100 [24].

For pain intensity, the scale is most anchored by “no pain” (score of 0) and “pain as bad as it could be” or “worst imaginable pain” (score of 100 on a 100-mm scale). The following cut points on the pain VAS will be used: no pain (0-4 mm), mild pain (5-44 mm), moderate pain (45-74 mm), and severe pain (75-100 mm) [25].

##### Electromyography

The EMG of the masseter and the temporalis will be recorded with the individuals seated with their heads guided in the Frankfurt horizontal plane. The masseter and temporal anterior muscles will be examined with surface electrodes at an interelectrode distance of 20 mm positioned on the muscle bellies parallel to the muscle fibers (“temporal anterior: vertically along the anterior muscular margin around the coronal suture; masseter: parallel to the muscle fibers, with the upper pole of the electrode at the intersection between the tragus-labial commissure and the exocanthion-gonion lines”) [26]. Reference electrodes will be applied in positions “inferior and posterior to the right ear” [27].

#### Secondary Outcome Measurements

##### Pain Drawing

The pain reported in distinct body regions, especially if related to known regional disorders (eg, headache, back pain, pelvic pain, or neck pain), can be summarized as a count variable. Extent of pain can be computed as percent of the body area [28].

##### Graded Chronic Pain Scale, Version 2.0

This scale [29] includes three items for pain intensity, four items for function, and one item for number of days of pain. *Characteristic pain intensity* is the computed mean of items 2 to 4 (pain right now, worst pain, average pain) multiplied by 10. The *interference score* is the computed mean of items 6 to 8 (daily activities, social activities, work activities) multiplied by 10. The total disability points are the sum of points for disability days + points for interference score. Based on the scores, chronic pain is graded as 0 (none), I (low-intensity pain, without disability), II (high-intensity pain, without disability), III (moderately limiting), and IV (severely limiting).

##### Jaw Functional Limitation Scale

This scale [30] with 20 items is used to calculate a single global score of “jaw functional limitation” by computing the mean of the available items. Subscale scores for each type of functional limitation are computed as follows:

- Mastication: mean of items 1 to 6
- Mobility: mean of items 7 to 10
- Verbal and nonverbal communication: mean of items 13 to 20

##### Oral Behaviours Checklist

Scoring can be computed as the sum of the number of items with nonzero response. Based on comparison of individuals with chronic TMD versus those without TMD, an Oral Behaviours Checklist summary score of 0 to 16 appears to represent normal behaviors, while a score of 17 to 24 occurs twice as often in those with TMD, and a score of 25 to 62 occurs 17 times more often. As a risk factor for TMD, only a score in the 25 to 62 range contributes to TMD onset [31].

##### Patient Health Questionnaire-9: Depression

The Patient Health Questionnaire (PHQ)-9 [32] is comprised of 9 items assessing depressed mood. A total sum score is computed. Scores of 5, 10, 15, and 20 represent cut points for mild, moderate, moderately severe, and severe depression, respectively.

##### Patient Health Questionnaire-15: Physical Symptoms

The PHQ-15 [33] is comprised of 15 items and assesses nonspecific physical symptoms, also referred to as functional symptoms or medically unexplained symptoms. Items are scored by adding the individual responses. A total sum score is computed. Scores of 5, 10, and 15 represent cut points for low, medium, and high physical symptoms, respectively.

##### General Anxiety Disorder-7: Anxiety

The General Anxiety Disorder-7 [34] is comprised of 7 items assessing anxious mood and behavior. A total sum score is computed. Scores of 5, 10, and 15 represent cut points for mild, moderate, and severe anxiety, respectively.

### Participation Timeline

**Figure 2:**
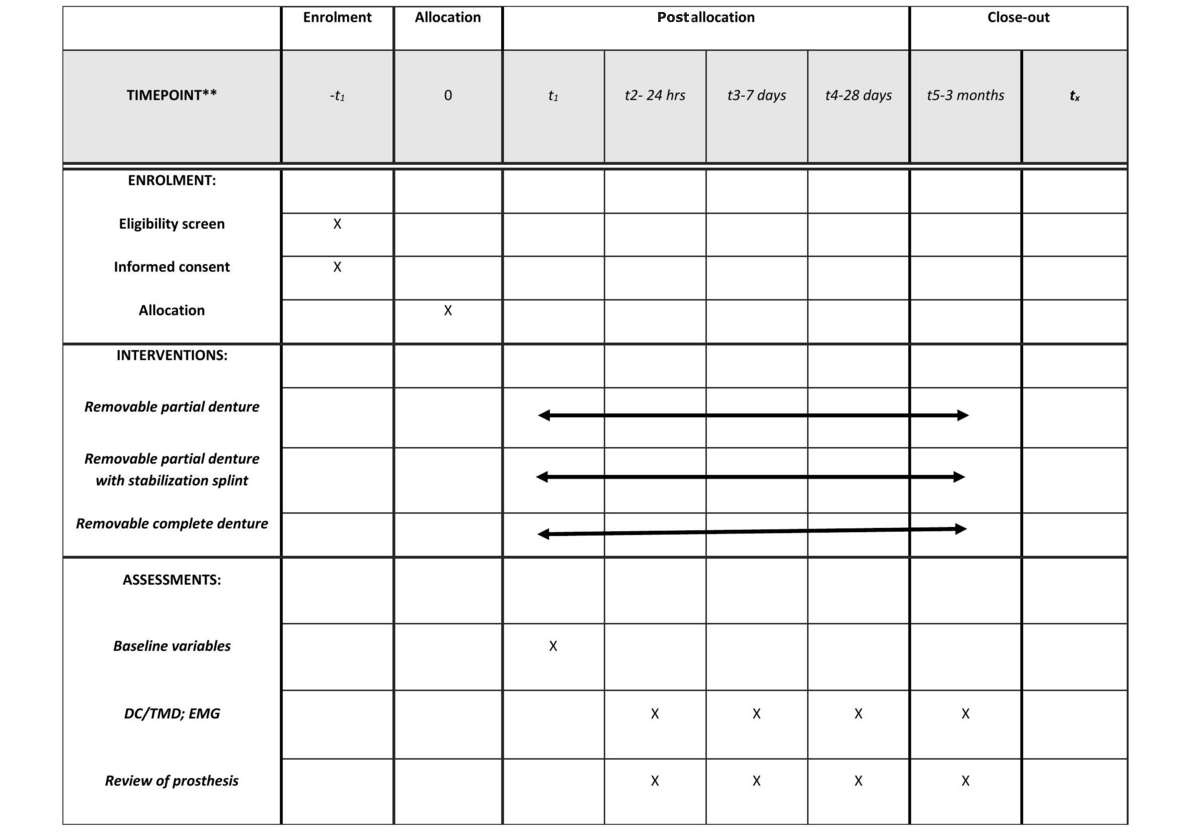
Schedule of enrollment, interventions, and assessment. DC/TMD: Diagnostic Criteria for Temporomandibular Disorders; EMG: electromyography.

Each eligible patient will be participating in the trial for 3 months, 1 year from their visit (baseline). The total number of visits will depend on the study arms to which the participants are allocated, but the outcome measurements will be recorded at 5 visits (baseline, 24 hours, 1 week, 28 days, and 3 months; Figure 2 and Table 1).

**Table 1:** Participation timelines.

Procedures		1^a^ (0 weeks)	2^b^ (2 weeks)	3 (24 hours)	4 (7 days)	5 (28 days)	6 (3 months)
Prescreening consent		Yes	No	No	No	No	No
Informed consent		Yes	No	No	No	No	No
Oral examination		Yes	No	No	No	No	No
Eligibility assessment		Yes	No	No	No	No	No
Allocation to study arms		Yes	No	No	No	No	No
**Prosthetic intervention**							
	Group 1	No	Yes	Yes	Yes	Yes	Yes
	Group 2	No	Yes	Yes	Yes	Yes	Yes
	Group 3	No	Yes	Yes	Yes	Yes	Yes
Compliance		Yes	No	No	No	No	No
Assessment of DC/TMD^c^, EMG^d^, review of prosthesis		Yes	Yes	Yes	Yes	Yes	Yes
Adverse event assessments		No	Yes	Yes	Yes	Yes	Yes

^a^It may be single or multiple based on patient-informed consenting process.

^b^It may be multiple based on the type of prosthesis being delivered.

^c^DC/TMD: Diagnostic Criteria for Temporomandibular Disorders.

^d^EMG: electromyography.

### Sample Size

The activity of masticatory muscle (mean and SD) while chewing, estimated by EMG, reflects the TMD in a patient [35]. With a power of 80%, alpha error of .05, and effect size of 0.77, the minimum sample size estimated for each group was 22. Considering attrition and the inclusion of various variables (edentulousness location, etc), we increased the sample to 50 for each interventional group. The sample size of 50 will have a minimum of 20 patients with edentulous maxilla and 20 patients with edentulous mandible for intervention groups 1 and 2. The total sample size for the study is 300 with 150 for the interventional group and 150 for the control group. Please refer to Table 2 for the sample breakdown.

**Table 2:** Sample size breakdown.

			Group 1: removable partial denture, n		Group 2: removable partial denture with stabilization splint, n		Group 3: removable complete denture, n		Total, n
**Intervention**			50		50		50		150
	Patients with edentulous maxilla	20		20		20			
	Patients with edentulous mandible	20		20		20			
	Total	40		40		40			
	Females	20		20		20			
	Males	20		20		20			
	Total	40		40		40			
**Control**			50		50		50		150
	Patients with edentulous maxilla	20		20		20			
	Patients with edentulous mandible	20		20		20			
	Total	40		40		40			
	Females	20		20		20			
	Males	20		20		20			
	Total	40		40		40			
Sample per group			100		100		100		300

### Recruitment

The adults visiting for routine dental care prosthodontics, Amrita School of Dentistry or at Amrita Urban Dental Health Centre will be recruited for the study. On examination, if the patient is found to satisfy the inclusion criteria, then they will be given options for the management of TMD by prosthetic rehabilitation or by splints. If the patient opts for prosthetic rehabilitation, then an option of participation in the trial will be explained, and the consenting process is followed. In case the patient declines to participate in the trial, the missing teeth will be managed according to the standard protocol of the department.

### Sequence Generation

The selected patient for trial will be randomly allocated to either the intervention arm or the control arm of their group, as per computer-generated randomization, schedule stratified, based on the type of group it belongs. The selected individuals will be allocated in equal probabilities to the intervention and control groups by stratified randomization. Stratified randomization is achieved by performing a separate randomization procedure within each of the two strata of participants. This will ensure that the numbers of participants receiving each intervention are closely balanced within each stratum. Treatment assignments are then made from separate randomization lists created in advance of the trial for each stratum.

### Allocation Concealment Mechanism

Allocation concealment will be ensured, the randomization code will not be released until the patient has been recruited into the trial, which takes place after all baseline measurements have been completed. The randomized codes, kept in a sealed cover at each clinic, will assign the patient to the intervention at baseline visit. The statistician will design the randomization schedule.

### Implementation

The randomization will be conducted by a statistician to manage the data, and the statistician will be blind against the study condition. The randomization list will remain with the doctoral committee for the whole duration of the study. Thus, randomization will be conducted without any influence of the dentist and hygienist.

### Blinding

Due to the obvious nature of the treatment, neither the treating dentist or patients are blinded; patients are only randomly allocated to the intervention. However, the data analyst or statistician will be blinded during the statistical process.

### Data Collection Methods

Each personnel will be trained centrally for the study requirements, standardization of examination and assessment of the outcomes and counselling for adherence, and the eliciting of information from study participants in a uniform reproducible manner. The data to be collected and the procedures to be conducted at each visit will be reviewed in detail. Each of the data collection forms and the nature of the required information will be discussed in detail on an item-by-item basis. Entering data forms, responding to data discrepancy queries, and general information about obtaining research quality data will also be covered during the training session. Once an individual is enrolled or randomized, the study site will make every reasonable effort to follow the individual for the entire study period of 12 months. It is projected that the rate of loss-to-follow-up on an annual basis will be at most 5%.

### Retention

Everyone has the right to withdraw from the trial at any time. In addition, the treating dentist may discontinue an individual from the trial at any time if they consider it necessary for any reason, including:

- Ineligibility (either arising during the trial or retrospectively having been overlooked at screening)
- Significant protocol deviation
- Significant noncompliance with treatment regimen or trial requirements
- An adverse event that requires discontinuation of the trial or results due to inability to continue to comply with trial procedures
- Disease progression that requires discontinuation of the trial or results due to inability to continue to comply with trial procedures
- Withdrawal of consent
- Loss of follow-up

Participants also may be withdrawn if the regulatory authorities terminate the study prior to its planned end date. If the participant is withdrawn due to an adverse event, the treating dentist will arrange for follow-up visits or telephone calls until the adverse event has resolved or stabilized.

### Compliance With Trial Treatment

The follow-up visits of the individual are important for the trial. Compliance to visit the dental clinic at designated intervals is done by telephonic and email reminders to the individuals. If the individual has missed the appointment or wants to reschedule, then it is rescheduled within +/– 2 days. If the individual misses two rescheduled appointments, then it is referred as noncompliance. The individual will be withdrawn from the study. However, if the individual completes 6 months and becomes noncompliant for the next 6 months, the person’s time of participation in the trial will be counted and will be used for analysis.

### Data Forms and Data Entry

This may be done at a center or at the participating site where the data originated. Original study forms will be entered and kept on file at the participating site. Participant files are to be stored in numerical order and stored in a secure and accessible place and manner. Participant files will be maintained in storage for a period of 3 years after completion of the study.

All forms related to study data will be kept in locked cabinets. Access to the study data will be restricted. All reports will be prepared such that no individual patient can be identified. The study sites will send 6-month email reports with information on missing data, missing forms, and missing visits.

### Statistical Methods

The effectiveness of pain reduction within each arm and between each group will be analyzed using analysis of variance. The details of the analysis are explained in Table 3.

**Table 3:** List of proposed analysis for trial results.^a^

Variable/outcome			Hypothesis		Outcome measure		Methods of analysis
Effectiveness of reduction of pain: within each arm			Intervention improved outcome from baseline to 12 months		Per person reduction of VAS^b^ score and GCPS^c^ (proportion or mean)		Chi-square methods or ANOVA^d^
Effectiveness of reduction of pain: between group 1, group 2, and group 3			Intervention improved outcome from baseline to 12 months		Per person reduction of VAS score and GCPS (proportion or mean)		Chi-square methods or ANOVA
**Subgroup analysis**							
	Effectiveness of reduction of pain: between group 1, group 2, and group 3	Intervention improved outcome from baseline to 12 months		Per person reduction of VAS score and GCPS (proportion or mean)		Regression methods with appropriate interaction term	
	Between posterior teeth rehabilitation vs anterior teeth rehabilitation	Number of teeth affects effectiveness of outcome		Per person reduction of VAS score and GCPS (proportion or mean)		Chi-square methods	
	Upper vs lower teeth	Position of edentulism affects the effectiveness outcome		Per person reduction of VAS score and GCPS (proportion or mean)		Chi-square methods	
	Duration of edentulism	Duration of edentulism affects pain outcome		Per person reduction of VAS score and GCPS (proportion or mean)		Chi-square methods	
	Number of quadrants	Number of quadrants affects the effectiveness outcome		Per person reduction of VAS score and GCPS (proportion or mean)		Chi-square methods	

^a^In all analyses, results will be expressed as coefficient, SEs, corresponding 95%, and associated *P* values. Goodness of fit will be assessed by examining the residuals for model assumptions and chi-square tests for goodness of fit.

^b^VAS: visual analog scale.

^c^GCPS: Graded Chronic Pain Scale.

^d^ANOVA: analysis of variance.

### Analysis Population and Missing Data

We will report reasons for withdrawal for each randomization group and compare the reasons qualitatively. The effect that any missing data might have on results will be assessed via sensitivity analysis of augmented data sets. Dropouts will be included in the analysis by using multiple imputation methods for missing data. Excluding patients from the analysis who violated the research protocol (did not get their intended treatment) can have significant implications that impact the results and analysis of a study. If the attrition loss is less than 15%, a per protocol analysis will be carried out; otherwise, “intention to treat” will be performed.

### Data Monitoring

An interim analysis is performed on the primary end point (reduction in pain) when 50% of patients have been randomized and have completed the 12-month follow-up. The interim analysis will be performed by an independent statistician, blinded for the treatment allocation.

### Research Ethics Approval

This clinical trial protocol has been approved by the Institutional Review Board (IRB) of Amrita Institute of Medical Sciences, Kochi, India. The investigator will ensure that this trial is conducted in accordance with the principles of the Declaration of Helsinki. The protocol, site-specific informed consent forms (speaking language), participant education and recruitment materials, and other requested documents—and any subsequent modifications—will be reviewed and approved by the IRB. The chief investigator will ensure that this trial is conducted in accordance with relevant regulations and with Good Clinical Practice. The chief investigator shall submit once a year throughout the clinical trial, or on request, an annual progress report to the IRB. In addition, an end of trial notification and final report will be submitted.

### Consent to Participate

A trained trial coordinator will introduce the trial to patients who will be shown a video regarding the main aspects of the trial. Patients will also receive information sheets. They will discuss the trial with patients considering the information provided in the video and information sheets. Patients will then be able to have an informed discussion with the participating consultant. The participant will be detailed on no less than the exact nature of the trial, what it will involve for the participant, the implications and constraints of the protocol, and the known side effects and any risks involved in taking part. It will be clearly stated that the participant is free to withdraw from the trial at any time for any reason without prejudice to future care, without affecting their legal rights, and with no obligation to give the reason for withdrawal.

They will obtain written consent from patients willing to participate in the trial. Information sheets and consent forms are provided for all participants involved in the trial. All information sheets, consent forms, and the video transcript will be in speaking language. Written and verbal versions of the participant information and informed consent will be presented to the patient. They will be allowed as much time as wished to consider the information and have an opportunity to question the treating dentist to decide whether they will participate in the trial. Written informed consent will then be obtained by means of a participant dated signature and dated signature of the person who presented and obtained the informed consent. A copy of the signed informed consent will be given to the participant. The original signed form will be retained at the trial site.

### Protocol Amendments

Any modifications to the protocol that may impact the conduct of the study or potential benefit of the patient or may affect patient safety, including changes of study objectives, study design, patient population, sample sizes, study procedures, or significant administrative aspects will be notified to the IRB for approval.

### Confidentiality

The trial staff will ensure that the participants’ anonymity is maintained. The participants will be identified only by a participant ID number on all trial documents and any electronic database. All documents will be stored securely and only accessible by trial staff and authorized personnel. The trial will comply with the Data Protection Act, which requires data to be anonymized as soon as it is practical to do so. Source documents are where data are first recorded and from which participants’ case report form (CRF) data are obtained. These include, but are not limited to, hospital records (from which medical history and previous and concurrent medication may be summarized into the CRF), clinical and office charts, laboratory and pharmacy records, diaries, radiographs, and correspondence.

CRF entries will be considered source data if the CRF is the site of the original recording (ie, there is no other written or electronic record of data). All documents will be stored safely in confidential conditions. On all trial-specific documents other than the signed consent, the participant will be referred to by the trial participant number/code, not by name. The participants will be identified by a unique trial specific number or code in any database.

## Results

Ethical approval was obtained from the IRB of Amrita Institute of Medical Sciences, Kochi, India. Informed consent will be obtained from all participants before recruiting to the study. Recruitment began in May 2021.

## Discussion

### Overview

There have always been controversies regarding occlusion as a causative factor for TMD. The effect of partial/total edentulism and their rehabilitation on temporomandibular joint has not been documented in any long-term clinical trials. To better understand this phenomenon, long-term studies that evaluate both edentulism as a cause of TMD and prosthetic rehabilitation as a prevention or cure of TMD must be conducted. Even though the prevalence of TMD in association with edentulism and in rehabilitated patients has been increasing, proper guidelines for the management of such cases have not been established. Any attempt to identify and symptomatically treat to relieve such patients from pain also means improvement of their quality of life.

### Strengths and Limitations of This Study

This study has the following strengths and limitations:

- The study design (RCT) will provide evidence of prosthetic rehabilitation on the TMD.
- Patients with TMD are grouped based on their edentulous state as completely or partially edentulous, and partially edentulous patients are classified further based on the Kennedy classification. This ensures that the effect of different types of edentulism on TMD are assessed.
- EMG reading for recording the muscle activity is an additional quantitative outcome evaluation used to assess and evaluate the TMD.
- As the study has a follow-up period of 3 months after the intervention, we expect some amount of attrition.

### Conclusion

This protocol has input from a study that we conducted on a tribal population to find out the prevalence of TMD. The study found out that there is a relation between the type of edentulism and TMD. The research question was developed based on this research experience and understanding that there is a lack of well-designed trials to evaluate the effects of prosthetic rehabilitation on TMDs. The study has been designed by grouping edentulous patients to different strata based on the types of edentulism.

## Appendix

Multimedia Appendix 1 Higher-resolution version of Figure 1.

## Abbreviations

CRF: case report form
DC/TMD: Diagnostic Criteria for Temporomandibular Disorders
EMG: electromyography
IRB: Institutional Review Board
PHQ: Patient Health Questionnaire
SPIRIT: Standard Protocol Items: Recommendations for Interventional Trials
TMD: temporomandibular disorder
VAS: visual analog scale
