# Supplementary figures and images for: Effects of Prosthetic Rehabilitation on Temporomandibular Disorders: Protocol for a Randomized Controlled Trial
Source: JMIR Res Protoc. 2021 Dec 24;10(12):e33104. doi: 10.2196/33104 (PMC8742205; doi:10.2196/33104)

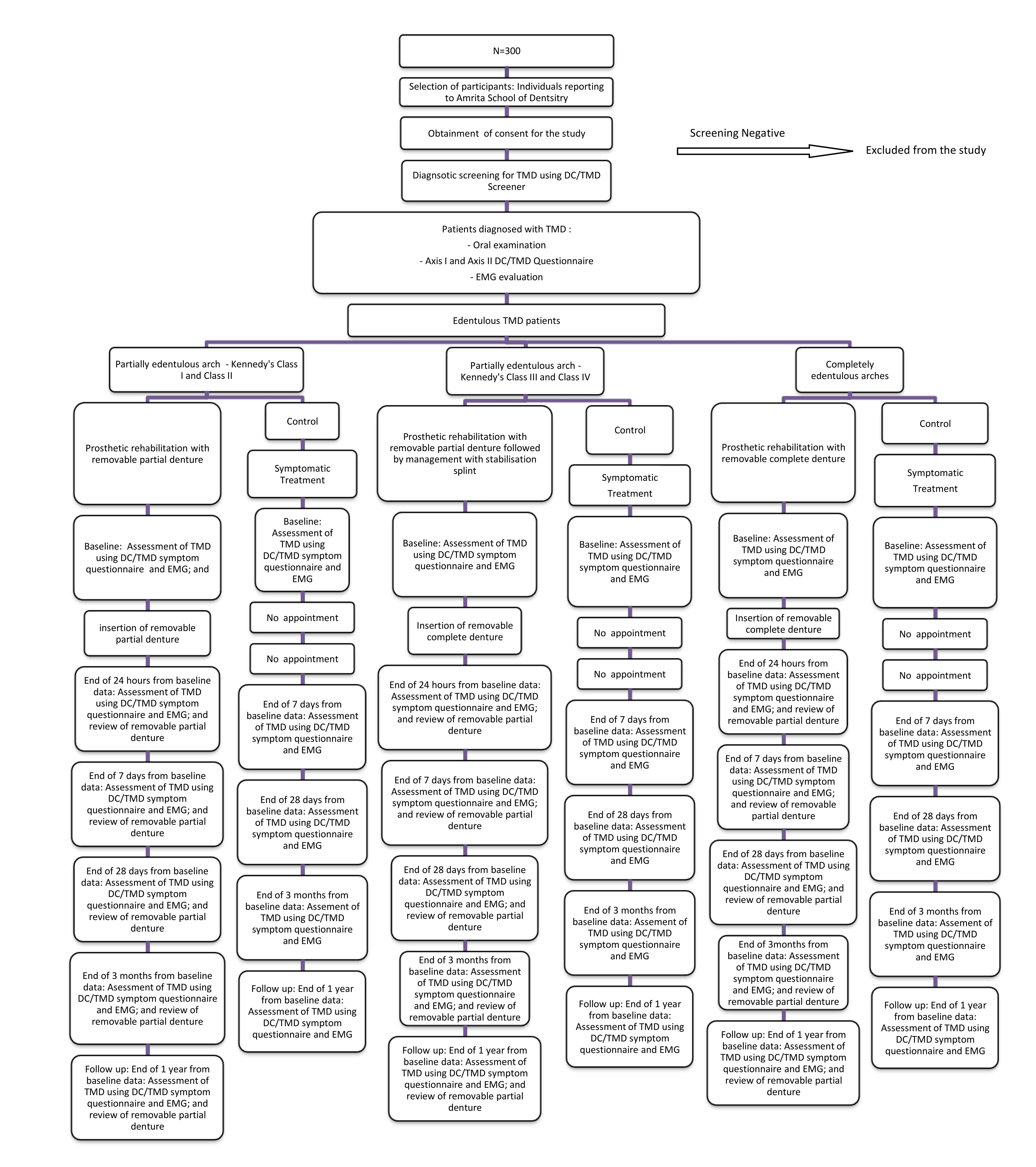

Supplement: Multimedia Appendix 1 [file resprot_v10i12e33104_app1.png]
